# Bottom‐Up Photosynthesis of an Air‐Stable Radical Semiconductor Showing Photoconductivity to Full Solar Spectrum and X‐Ray

**DOI:** 10.1002/advs.202302978

**Published:** 2023-08-04

**Authors:** Yu Zhang, Yun‐Fan Yan, Jia‐Rong Mi, Shuai‐Hua Wang, Ming‐Sheng Wang, Guo‐Cong Guo

**Affiliations:** ^1^ College of Chemistry Fuzhou University Fuzhou Fujian 350108 P. R. China; ^2^ State Key Laboratory of Structural Chemistry Fujian Institute of Research on the Structure of Matter Chinese Academy of Sciences Fuzhou Fujian 350608 P. R. China

**Keywords:** halides, photoconductivity, photosynthesis, radical, semiconductors

## Abstract

Single‐component semiconductors with photoresponse to full solar spectrum are highly desirable to simplify the device structure of commercial photodetectors and to improve solar conversion or photocatalytic efficiency but remain scarce. This work reports bottom‐up photosynthesis of an air‐stable radical semiconductor using BiI_3_ and a photochromism‐active benzidine derivative as a photosensitive functional motif. This semiconductor shows photoconductivity to full solar spectrum contributed by radical and non‐radical forms of the benzidine derivative. It has also the potential to detect X‐rays because of strong X‐ray absorption coefficient. This finding opens up a new synthetic method for radical semiconductors and may find applications on extending photoresponsive ranges of perovskites, transition metal sulfides, and other materials.

## Graphical abstract

An air‐stable single‐component radical semiconductor with photoresponse to full solar spectrum and X‐ray is prepared by a bottom‐up photosynthetic method using BiI_3_ and a photochromism‐active benzidine derivative as a photosensitive functional motif.

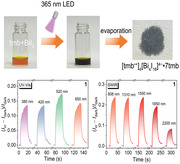

## 1 Introduction

Photoresponsive range is a key parameter of semiconductors for solar conversion, photocatalysis, and photodetection.^[^ ^1^, ^2^ ^]^ The photoresponsive upper limit of monocrystalline or polycrystalline silicon in commercial solar cells is usually less than 1100 nm,^[^ ^3^ ^]^ and the rest of the full solar spectrum (≈295–2500 nm) is still not effectively used. As for photocatalysis, the majority of single‐component photocatalysts have limited photoresponsive ranges in the visible spectrum. For instance, the photoresponsive range of the well‐known photocatalyst TiO_2_ is out of the visible spectrum. The limited range usually leads to low photocatalytic efficiency.^[^ ^4^ ^]^ To realize photodetection for the full solar spectrum, a general commercial structure is the integration of two detectors with complementary photoresponsive ranges, such as Si (≈200–1100 nm) and InGaAs (≈900–1700 nm) photodetectors. This structure makes the system very complex and sometimes difficult to maintain. Therefore, the exploration of semiconductive materials with photoresponse to the full solar spectrum has been an important aim in the fields of solar utilization, photocatalysis, and photodetection.

There have been many traditional methods to broaden the photoresponsive ranges of semiconductors, such as doping,^[^ ^5^ ^]^ surface coating,^[^ ^6^ ^]^ introducing a component with localized surface plasmon resonance effect,^[^ ^7^ ^]^ embedding defects,^[^ ^8^ ^]^ and forming a quantum well structure.^[^ ^9^ ^]^ Among these methods, doping and surface coating using a photosensitizer are the most common two ones. However, the broadened ranges are far from covering the full solar spectrum. For example, the absorption upper limit of Zn_x_Cd_1−x_S is about 900 nm.^[^ ^10^ ^]^ Integration of two or more semiconductive components with complementary photoresponsive ranges is a novel and effective method to achieve photoresponse to the full solar spectrum.^[^ ^11^ ^]^ For instance, Yu and co‐workers united three semiconducting sulfides, namely ZnS, CdS, and Cu_2−x_S, into a single nanocrystal through a colloidal chemical transformation strategy and realized full harvest of solar energy.^[^ ^12^ ^]^ One shortage of this multi‐component method is the complex preparation process.

Except for the above work, some efforts have been made to achieve single‐component semiconductors with intrinsic photoresponsive ranges covering the full solar spectrum. To date, very few of these semiconductors have been found. The representatives include graphene,^[^ ^13^ ^]^ black phosphorus/phosphide,^[^ ^14^ ^]^ and crystalline topological insulators (for example, SnTe).^[^ ^15^ ^]^ They have some deficiencies need to be addressed before their use in applications. For instance, graphene has low absorption coefficient (<3%) and high dark current; black phosphorus/phosphide is not stable in air; crystalline topological insulators are still limited by poor crystal quality and unstable morphology.^[^ ^1^ ^]^ In recent years, we synthesized some single‐component photochromic semiconductors based on viologen and analogues.^[^ ^16^, ^17^, ^18^, ^19^, ^20^ ^]^ They undergo photoinduced electron transfer and yield stable radical products with broadband absorption. For instance, a series of 2D cyanide‐bridged semiconductor with infinitely π‐stacked redox‐active *N*‐methyl bipyridinium cations were prepared through layer‐directed intercalation approach.^[^ ^16^ ^]^ Their photoinduced radical products showed photoresponse to the full solar spectrum. Such post‐photosynthetic methods are still subject to the low content of photogenerated radicals and limited improvement of photoelectric properties because of the low penetration of common lights inside the crystals.

**Figure 1:**
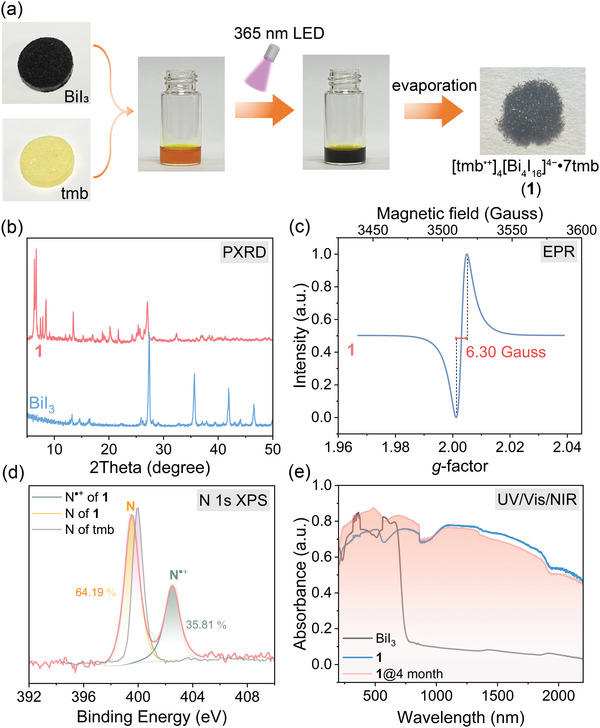
Photosynthesis and radical feature of **1**. a) The photosynthetic process. b) Powder X‐ray diffraction (PXRD) patterns of **1** and BiI_3_. c) EPR spectrum of **1**. d) N 1s XPS data of **1** and tmb. e) UV/vis/NIR absorption spectra of BiI_3_ and **1** before and after standing in air for 4 months.

In this work, we develop an unprecedented bottom‐up photosynthetic method to obtain single‐component semiconductors with photoresponse to the full solar spectrum. The key is to introduce a photosensitive functional motif with broadband absorption in the solar spectrum. Chopoorian et al. reported in 1964 that *p*‐phenylenediamine tetraacetic acid absorbed in porous glass showed photochromism.^[^ ^21^ ^]^ A radical product was observed after irradiation. It was ascribed to photoinduced electron transfer from the organic component to the glass, that is, *p*‐phenylenediamine tetraacetic acid was oxidized. The Gopidas group^[^ ^22^ ^]^ and the Wang group,^[^ ^23^ ^]^ respectively, found that oxidation of benzidine and its derivatives by Cu(ClO_4_)_2_ and silver(Ι) salts yielded radical cations with two absorption bands around 470 and 1000 nm. We here found, for the first time, that direct irradiation of *N,N,N’,N’*‐tetramethylbenzidine (tmb) and BiI_3_ in a mixture of DMF and DMA yielded an air‐stable radical semiconductor [tmb^•+^]_4_[Bi_4_I_16_]^4−^⋅7tmb (**1**; **Figure** **1**a). This compound has strong absorption spanning the full solar spectrum. In comparison with BiI_3_, it has a more than 1750 nm redshift of the absorption edge and a conductivity gain of five orders of magnitude. Meanwhile, it shows photoconductivity to the full solar spectrum or X‐ray. To our knowledge, only a few single component detectors have been reported to exhibit both infrared and X‐ray response simultaneously (Table S2, Supporting Information).

## 2 Results and Discussions

When tmb and BiI_3_ was dissolved in the mixture of DMF and DMA, a clear orange solution was obtained (Figure 1a). The color of the solution gradually deepened upon irradiation of a 365 nm LED lamp (1.02 W cm^–2^) in air and reached saturation at 15 min (Figure S1, Supporting Information). Meanwhile, the UV/vis/NIR absorption intensity between 400 and 1600 nm were enhanced with increasing irradiation time (Figure S2, Supporting Information). The BiI_3_ solution shows an absorption band ranging from 300 to 600 nm while the tmb solution has an absorption band below 385 nm. We found that, unlike the 365 nm lamp, irradiation using a 420 nm lamp did not result in any color change (Figure S3, Supporting Information). Therefore, photoreaction of the BiI_3_ and tmb solution is initiated after photoexcitation of tmb. A control experiment revealed that O_2_ was required for this reaction (Figure S4, Supporting Information). Black plate single crystals were obtained after evaporation of the black solution. The characteristic IR peaks of **1** are consistent with those of tmb, which proves the presence of tmb in **1** (Figure S5, Supporting Information). They had a completely different powder X‐ray diffraction pattern to that of BiI_3_ (Figure 1b) and exhibited a single line signal at 3523 Gauss with *g* = 2.0030 and a linewidth of 6.30 Gauss in the electron paramagnetic resonance (EPR) spectrum (Figure 1c). This *g* value is close to 2.0023 for free electrons, which indicates the generation of a radical product. In addition, an X‐ray photoelectron spectroscopy (XPS) study (Figure 1d) showed that binding energies of both Bi and I elements are almost the same in BiI_3_ and **1** (Figure S6, Supporting Information), but that of the N element changed remarkably. The N 1s core‐level spectrum of the tmb molecule had a peak centered at 400.0 eV, while those of the black plate single crystals were split into two peaks at 399.6 and 402.5 eV, respectively. The appearance of the peak at 402.5 eV manifests the oxidation of the tmb molecule. Therefore, the black plate single crystals belong to a species containing the tmb^•+^ radicals.

**Figure 2:**
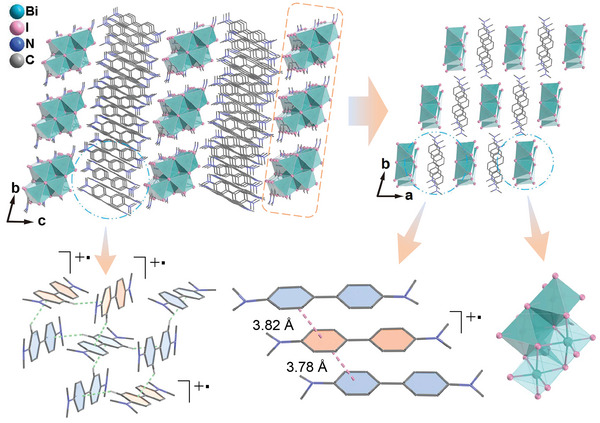
Crystal structure of **1**. C–H⋅⋅⋅π and π–π interactions are shown in green and purple dash lines, respectively. Only H atoms included in the C–H⋅⋅⋅π interactions are shown. All possible tmb^•+^ radicals are noted.

Electron absorption band of these crystals covers the full solar spectrum and is more than 1750 nm wider than that of BiI_3_ (Figure 1e). It is composed of three main sub‐bands at ≈500, 750, and 1200 nm, respectively.^[^ ^22^, ^23^ ^]^ The 500 nm band is contributed by the tmb^•+^ radical and the inorganic component. The 750 nm sub‐band and the shoulders at around 1500 and 2000 nm in the 1200 nm sub‐band are different from those of BiI_3_ (Figure 1e) and the reported radical cations of benzidine and its derivatives.^[^ ^22^, ^23^ ^]^ The clear red‐shift nature of the electron absorption spectrum compared with that of the tmb^•+^ radical and the π‐stacking structure (described below; **Figure** **2**) indicate the contribution of intervalent charge‐transfer transition between the neutral molecule and the tmb^•+^ radical. The absorption band almost remained when the sample was placed in the dark in air for 4 months (Figure 1e), which suggests a high air stability of the radical product.

In the crystal structure, compound **1** consists of one tetranuclear [Bi_4_I_16_]^4−^ cluster and eleven tmb units per molecule. The tetranuclear cluster is built by edge‐sharing of four BiI_4_^−^ ions (Figure 2). To satisfy the valence balance, four tmb units should be oxidized to form the tmb^•+^ cation radical. The XPS data shown in Figure 1d revealed that the area ratio of two N 1s peaks is 0.56, which agrees well the presence of 4 tmb^•+^ cation radicals and 7 neutral tmb units per molecule of **1**. C, H, and N contents are also in accordance with the formula of [tmb^•+^]_4_[Bi_4_I_16_]^4−^⋅7tmb. A comparison of structures between organic units in **1** and optimized neutral tmb molecule and the tmb^•+^ radical cation (at the B3LYP/6‐31g** level; Figure S7, Supporting Information) figures out the 4 possible tmb^•+^ cation radicals (Figure 2). Three tmb units form a [tmb⋅⋅⋅tmb^•+^⋅⋅⋅tmb] trimer united by offset π–π stacking interactions. Stacking sequentially of this trimer and the [Bi_4_I_16_]^4−^ cluster along the *a* axis generates a chain, which is further stacked along the b axis to form a layer (Figure 2). The other 8 tmb units, including 5 neutral tmb molecules and 3 tmb^•+^ radicals, are intercalated between these layers and connected by C–H⋅⋅⋅π interactions (mean separation between centroid and H, 2.84 Å).

**Figure 3:**
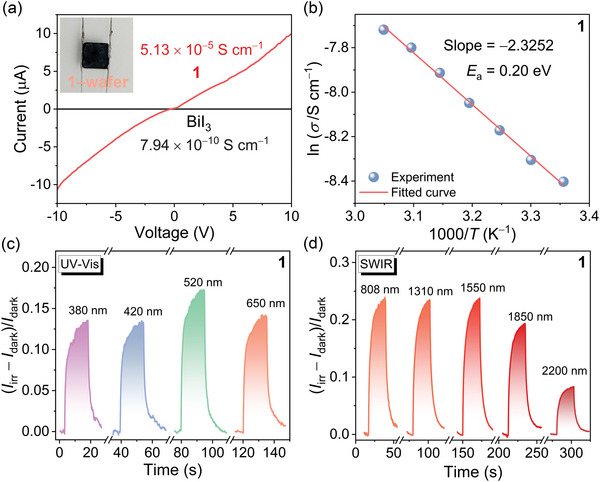
Electrical properties of **1**. a) Current–voltage (*I*–*V*) plot. Inset: Ag/**1**‐wafer/Ag device. The plot for BiI_3_ is also shown for comparison. b) Correlation between conductivity and temperature, fitted by the Arrhenius equation. c,d) Wavelength‐dependent photoelectric response using a Xe lamp (bias, 15 V) and a series of semiconductor lasers (bias, 5 V) for the UV–vis and SWIR regions, respectively. Note: the relative magnitude of the current change is not comparable because of the different powder densities of light.

To investigate the electrical behavior of **1**, a polycrystalline wafer (thickness, 0.18 mm) was used to construct Ag/**1**/Ag device (**Figure** **3**a, inset). As shown in Figure 3a, the dark current curve under continuous voltage (*I*–*V*) of the device manifested nearly linear relation, revealing its inherent Ohmic characteristics. The Wang group previously found that the benzidine radical cation–OC(CF_3_)_3_^−^ salt was nonconductive.^[^ ^23^ ^]^ In contrary, compound **1** has a conductivity of 5.13 × 10^−5^ S cm^−1^, which is comparable to that of GaAs and is also five orders of magnitude higher than that of the BiI_3_ precursor (7.94 × 10^−10^ S cm^−1^). Compound **1** is stable after thermal annealing at 110 °C for 1 h. However, it has a phase transition at around 130 °C (Figure S8, Supporting Information), and starts to decompose at ≈200 °C (Figure S9, Supporting Information). The conductivity increased with rising temperature between 20 and 60 °C (Figure 3b) and a linear trend was well fitted using the Arrhenius equation (ln*σ* = −*E*_a_/*k*_B_ *T* + constant), where *k*_B_ is the Boltzmann constant and *T* is the absolute temperature, respectively.^[^ ^24^ ^]^ The large charge density was derived from the low activation energy (*E*_a_ = 0.20 eV), which was in accordance with its intrinsically high conductivity. Figure 3c,d illustrates photoelectric response of **1** using a Xe lamp and a series of semiconductor lasers for the UV–vis (380, 420, 520, 650 nm) and shortwave infrared (SWIR: 808, 1310, 1550, 1850, 2200 nm) regions, respectively, was recorded to validate photodetection performance in the full solar spectrum. The relative magnitude of the current change between dark (*I*_dark_) and bright (*I*_irr_) conditions, (*I*_irr_ − *I*_dark_)/*I*_dark_, was used to deduce photocurrent gain. As can be seen, the device unveiled a photocurrent gain upon irradiation of both the UV–vis and the SWIR light. For instance, the gains are about 15% at 650 nm (89.2 mW cm^−2^) and 24% at 1550 nm (324.8 mW cm^−2^).

**Figure 4:**
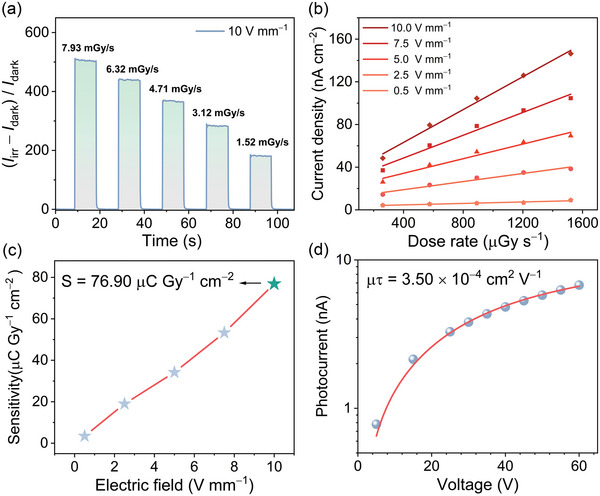
X‐ray detection performance of **1**. a) Photocurrent gain at various dose rates with an electric field of 10 V mm^−1^. b) Photocurrent density at different X‐ray dose rate and electric field. c) Sensitivity under different electric fields. d) Bias‐dependent photoconductivity.

Except the full solar spectrum, the Ag/**1**/Ag device shows also photoconductivity to X‐rays. The presence of the high atomic number elements Bi and I give **1** both a high density and a strong absorption coefficient to X‐ray. The calculated absorption coefficient of **1** is basically larger than that of Si in the 1–100 keV (Figure S10, Supporting Information). As shown in **Figure** **4**a, the device proved an evident response to the tungsten anode X‐ray, giving a ON/OFF ratio ranging from 180 to 500 with the dose rate rising from 1.52 to 7.93 mGy s^−1^. The sensitivity (*S*) of a detector reflects the charge collection efficiency under continuous X‐ray photons irradiation.^[^ ^25^ ^]^ As illustrated in Figure 4b,c, the photocurrent density and *S* value for the Ag/**1**/Ag device were enhanced with a raised electric field. At an electric field of 10 V mm^−1^, the *S* value was 76.90 μC Gy^−1^ cm^−2^, which was higher than that of the commercial Si detector.^[^ ^26^ ^]^ Furthermore, the mobility–lifetime (*μτ*) product represents the potential of a detector to extract X‐ray induced carriers.^[^ ^27^ ^]^ The calculated *μτ* value using the modified Hecht equation is 3.50 × 10^−4^ cm^2^ V^−1^ (Figure 4d).^[^ ^28^ ^]^ This value is comparable to that of MAPbI_3_ wafer (3.84 × 10^−4^ cm^2^ V^−1^).^[^ ^29^, ^30^ ^]^ Signal‐to‐noise ratio (SNR), evaluated under different bias, is a standard for detecting the amplitude of noise current and low detection limit (LoD).^[^ ^31^ ^]^ The minimum dose rate of our X‐ray detection system is 0.26 mGy s^−1^. At this dose rate, the SNR index for the Ag/**1**/Ag device was 70.93, 97.44, and 135.91 when applying bias voltages of 1, 5, and 15 V, respectively (Figure S11, Supporting Information). They are far larger than 3, indicating that the actual detection limit of the detector should be much smaller.

## 3 Conclusion

In summary, this work develops an unprecedented bottom‐up photochemical method to prepare radical semiconductors using photosensitive organic components. With the photochemical reaction of BiI_3_ and the photochromism‐active benzidine derivative, an air‐stable single‐component radical semiconductor was successfully synthesized. In comparison with BiI_3_, the electron absorption band and conductivity are significantly widened and enhanced, respectively. Especially, this radical semiconductor shows photoresponse to full solar spectrum and X‐ray. It may find applications for broadband photodetection, solar conversion or photocatalysis.

## 4 Experimental Section

### Measurements

Powder X‐ray diffraction (PXRD) patterns were collected on a Rigaku Desktop MiniFlex II diffractometer under a range from 5° to 50°, using Cu *K*_α_ (*λ* = 1.5406 Å) as radiation source powered at 30 kV and 15 mA. Analysis spectra of C, H, and N contents were recorded on an Elementar Vario MICRO microanalyzer. Thermogravimetric analysis (TGA) data were taken on a METTLER TOLEDO apparatus from 25 to 1000 °C under N_2_ at a heating rate of 10 °C min^−1^. Electronic absorption (UV/vis/NIR) spectra were recorded in the diffuse reflectance mode on a PerkinElmer Lambda 900 UV/vis/NIR spectrophotometer with a BaSO_4_ plate as the reference. FT‐IR spectra were determined by using a Perkin–Elmer spectrum instrument in the range of 4000−400 cm^−1^. Electron paramagnetic resonance (EPR) signals at the X band were recorded in a Bruker‐BioSpin E500 spectrometer at room temperature. X‐ray photoelectron spectroscopy (XPS) spectra were obtained with a ThermoFisher ESCALAB 250Xi X‐ray photoelectron spectrometer using Al *K*_α_ radiation (*λ* = 8.357 Å). X‐ray electricity studies were conducted on a Keithley 2400 instrument with a tungsten target continuous X‐ray spectrum light source. Routine electricity tests were executed in a Keithley 4200‐SCS semiconductor parameter analyzer with a PLS‐SXE300D 50‐W xenon lamp and a multi‐wavelength laser system. All pellet samples for tests were prepared by a two‐probe method using silver paste. For xenon lamp irradiation, bias was set as 15 V. Power density at each wavelength: 380 nm, 90.8 mW cm^−2^; 420 nm, 89.2 mW cm^−2^; 520 nm, 127.4 mW cm^−2^; 650 nm, 89.2 mW cm^−2^. For laser system irradiation, bias was set as 5 V. Power density at each wavelength: 808 nm, 331.2 mW cm^−2^; 1310 nm, 312.1 mW cm^−2^; 1550 nm, 324.8 mW cm^−2^; 1850 nm, 293.0 mW cm^−2^; 2200 nm, 165.6 mW cm^−2^. The detection area of optical power meter was estimated as 3.14 cm^2^ and the detection distance was fixed at 10 cm.

### Single‐Crystal X‐Ray Crystallographic Study

Single‐crystal X‐ray diffraction measurement of **1** was implemented at 100 K on Rigaku FR‐X Microfocus diffractometer (45 kV, 66 mA), using Cu‐*K*_α_ radiation (*λ* = 1.54178 Å). Intensity data collection and reduction were fulfilled by utilizing the CrysAlisPro software, and absorption correction was acquired by the multi‐scan method.^[^ ^32^ ^]^ The single crystal structure was solved by the direct method using Olex 2.1.5 and then refined by full‐matrix least‐squares method on *F* ^2^ via the Siemens SHELXTL software.^[^ ^33^, ^34^ ^]^ All hydrogen atoms were geometrically added and optimized using a riding model. No higher symmetry of the crystal structure was verified by PLATON. Crystallographic parameters were listed in Table S1 (Supporting Information). The entry of CCDC‐2253204 contains the supplementary crystallographic data for **1**. These data can be obtained free of charge at http://www.ccdc.cam.ac.uk/conts/retrieving.html or from the Cambridge Crystallographic Data Centre, 12, Union Road, Cambridge CB2 1EZ, U.K. Fax: (Internet) +44‐1223/336‐033. E‐mail: deposit@ccdc.cam.ac.uk.

### Syntheses

All chemicals and reagents were purchased commercially and directly used without further purification. BiI_3_ (0.05 mmol, 29.5 mg) and tmb (0.10 mmol, 24.0 mg) were dissolved in a mixture of 4 mL *N,N*‐dimethylformamide (DMF) and 4 mL *N,N*‐dimethylacetamide (DMA). The yielded solution was placed in air during a 365 nm LED irradiation (power density at 1.02 W cm^−2^) and black plate crystals of **1** was attained during the volatilization process. Yield based on BiI_3_: 35% for **1**. All crystalline samples for tests were hand‐picked under microscope. Anal. Calcd (%) for C_176_H_220_Bi_4_I_16_N_22_: C, 38.36; H, 4.02; N, 5.59; Found: C, 39.10; H, 3.98; N, 5.72.

### Structural Optimization

All structural optimizations were performed with the density functional theory (DFT) method at the B3LYP/6‐31g** level using the Gaussian 09 software package.^[^ ^35^ ^]^

### Charge Carrier Mobility‐Lifetime Product (*μτ*) Calculation

The *μτ* product was derived from bias‐dependent photoconductivity curve using a modified Hecht equation: where *I*_0_ is the saturated photocurrent, *L* is the wafer thickness, *V* is the applied bias, *τ* is the carrier lifetime.

(1) $$I=\frac{I_{0}\mu \tau V}{L^{2}}\left[1-\exp\left(\frac{-L^{2}}{\mu \tau V}\right)\right]$$

## Conflict of Interest

The authors declare no conflict of interest.

## Supporting information

Supporting Information

## Data Availability

The data that support the findings of this study are available from the corresponding author upon reasonable request.
