# Supplementary material for: Bottom‐Up Photosynthesis of an Air‐Stable Radical Semiconductor Showing Photoconductivity to Full Solar Spectrum and X‐Ray
Source: Adv Sci (Weinh). 2023 Aug 4;10(28):2302978. doi: 10.1002/advs.202302978 (PMC10558663; doi:10.1002/advs.202302978)
Supplement: Supplementary file 1 — Supporting Information [file ADVS-10-2302978-s001.pdf]

ADVANCED SCIENCE
Open Access

Supporting Information

for *Adv. Sci.*, DOI 10.1002/advs.202302978

Bottom-Up Photosynthesis of an Air-Stable Radical Semiconductor Showing Photoconductivity to Full Solar Spectrum and X-Ray

Yu Zhang, Yun-Fan Yan, Jia-Rong Mi, Shuai-Hua Wang, Ming-Sheng Wang and Guo-Cong Guo**

Supporting Information

Bottom-up Photosynthesis of an Air-Stable Radical Semiconductor Showing Photoconductivity to Full Solar Spectrum and X-ray

Yu Zhang[a, b], *Yun-Fan Yan*[b], *Jia-Rong Mi*[b], *Shuai-Hua Wang*[b], *Ming-Sheng Wang**[b], *and Guo-Cong Guo**[b]

a. College of Chemistry, Fuzhou University, Fuzhou, Fujian 350108 (P. R. China)

b. State Key Laboratory of Structural Chemistry, Fujian Institute of Research on the Structure of Matter, Chinese Academy of Sciences, Fuzhou, Fujian 350608 (P. R. China)

* Corresponding authors: mswang@fjirsm.ac.cn and gcguo@fjirsm.ac.cn

Additional Figures and Tables

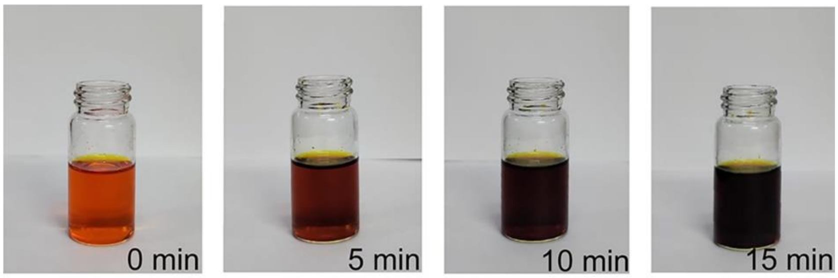

Figure S1. Photos of the reaction solution at different irradiation time.

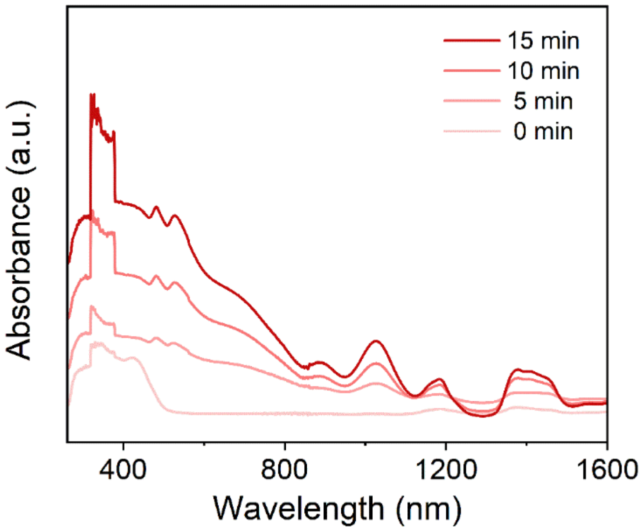

Figure S2. UV/Vis/NIR spectra of the reaction solution upon successive irradiation of the 365nm lamp.

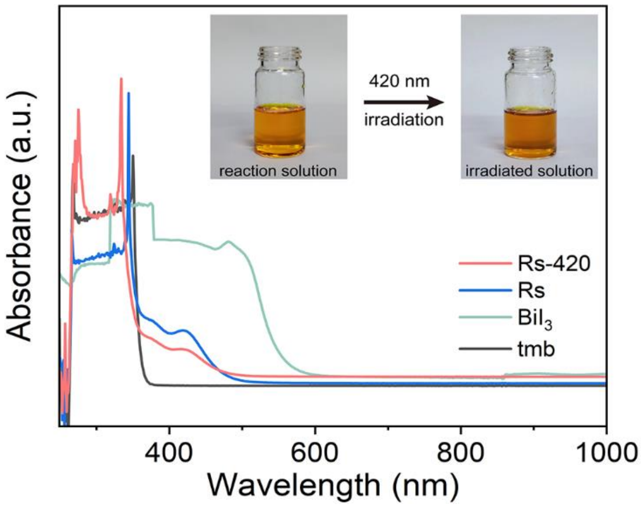

Figure S3 . UV/Vis spectra of reaction solution (Rs) before and after irradiation of the 420 nm lamp, as well as tmb and BiI_3 solution.

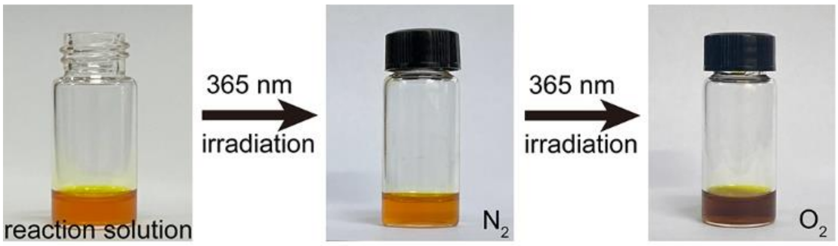

Figure S4. A control experiment of photoreactions between tmb and BiI_3 in DMF/DMA under different atmosphere.

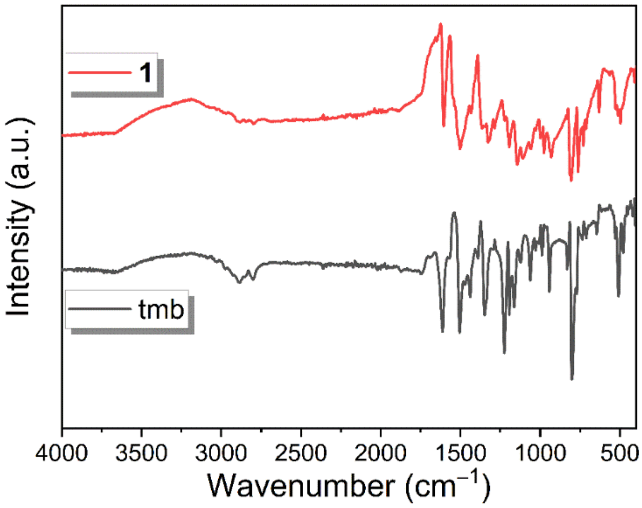

Figure S5. FT-IR spectra of **1** and tmb.

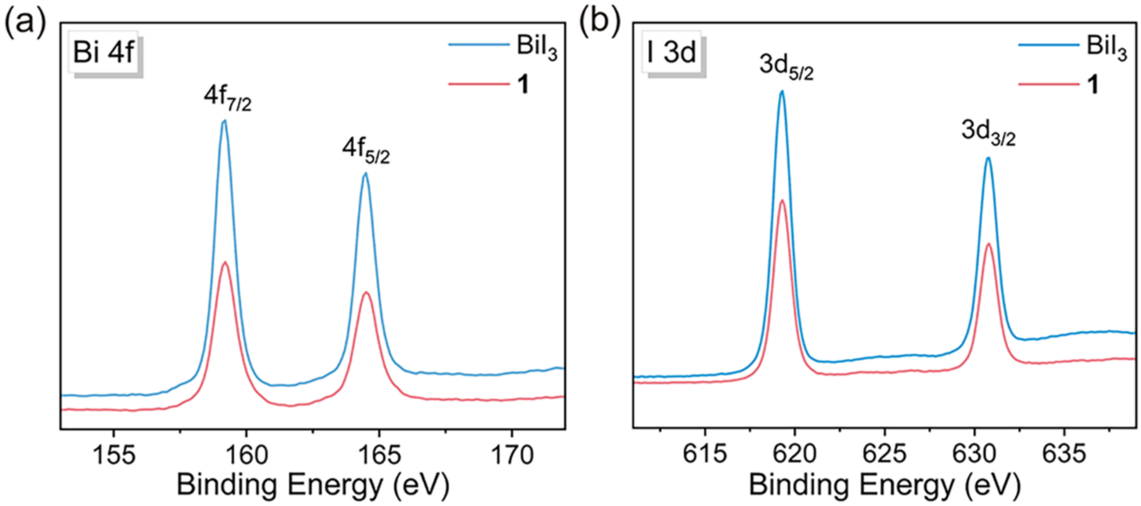

Figure S6. XPS data of Bi and I elements for **1** and BiI_3.

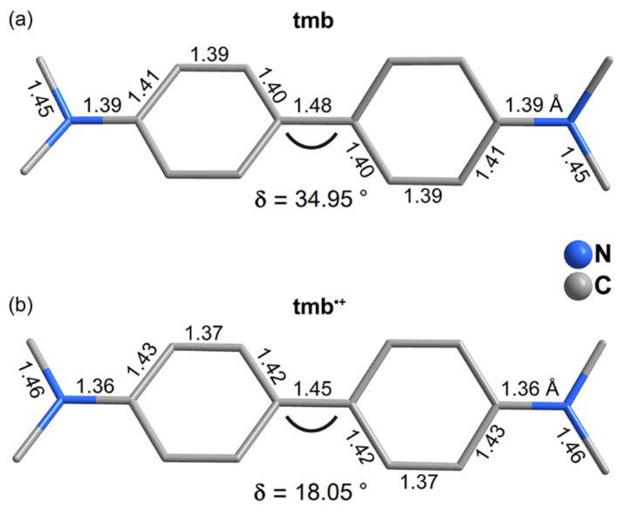

Figure S7. Calculated bond lengths and dihedral angles (δ) in optimized neutral tmb and tmb$^{\bullet+}$ molecules.

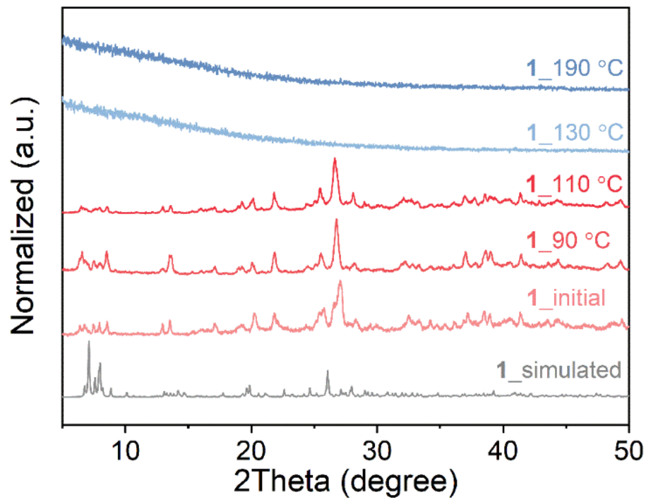

Figure S8. PXRD patterns of **1** upon thermal annealing at different temperatures. The simulated pattern is also included for comparison.

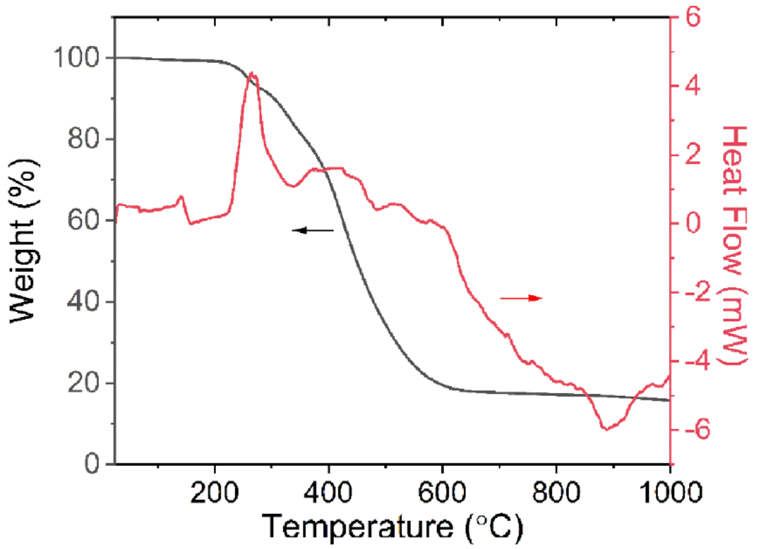

Figure S9. Thermogravimetric and differential scanning calorimetric (TG/DSC) data of **1**.

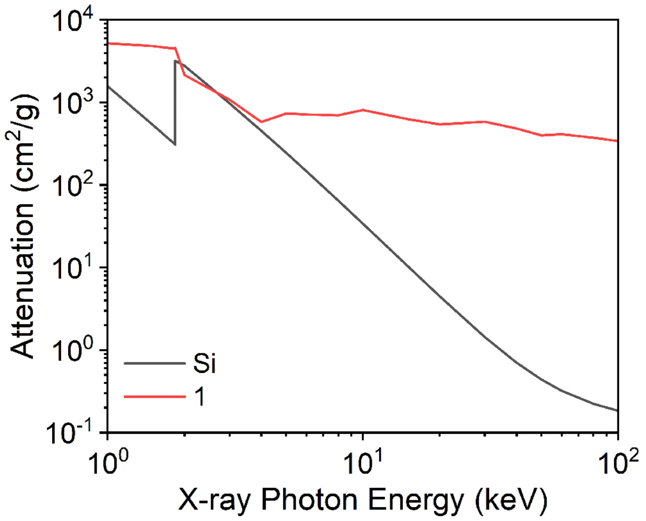

Figure S10. Attenuation coefficient of **1** and Si.

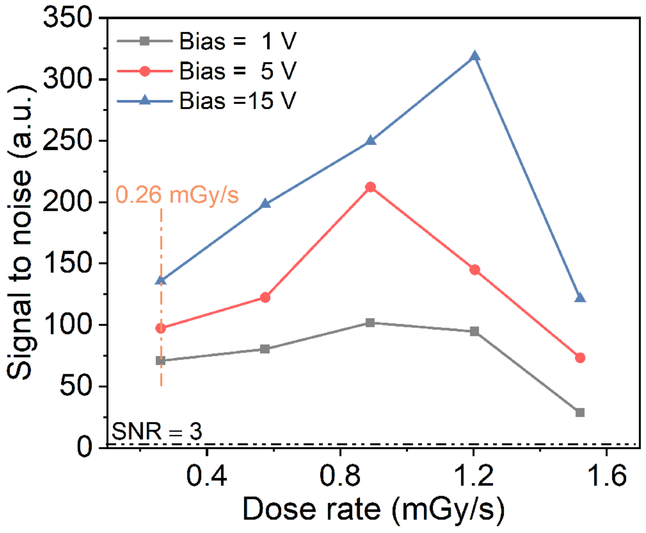

Figure S11. Signal-to-noise ratio (SNR) versus X-ray dose rate of **1** under various applied bias.

Table S1. Crystal and structure refinement data for **1**.

Formula	$C_{176}H_{220}Bi_4I_{16}N_{22}$
M_r	5510.05
Crystal size (mm^3)	0.28 × 0.15 × 0.03
Crystal system	Triclinic
Space group	*P*1
a (Å)	13.5867(2)
b (Å)	14.5343(3)
c (Å)	25.4486(4)
α (deg)	78.7610(10)
β (deg)	86.0720(10)
γ (deg)	65.845(2)
V (Å^3)	4497.11(15)
D_{calcd} (g/cm^3)	2.035
Z	1
F(000)	2610.0
Abs coeff (mm^{-1})	29.534
Reflns collcd/unique (R_{int})	93243/33367 (0.0438)
Data/params/restraints	33367/3/2008
Final *R* indexes [$I >= 2\sigma(I)$]	R_1[a] = 0.0418, ωR_2[b] = 0.1191
Final *R* indexes (all data)	R_1[a] = 0.0434, ωR_2[b] = 0.1205
GOF on F^2	1.028
Flack x	0.090(6)
$\Delta\rho_{max}$ and $\Delta\rho_{min}$ (e/Å^3)	1.68 and –2.35

[a] $R_1 = \sum||F_o|-|F_c||/\sum|F_o|$, [b] $\omega R_2 = \{\sum\omega[(F_o)^2-(F_c)^2]^2/\sum\omega[(F_o)^2]^2\}^{1/2}$.

Table S2. Responsive range comparison between **1** and reported detectors.

Materials	Modification methods	Responsive range		Ref.
		UV–NIR (nm)	X-ray	
BP/Si_xN_y	doping	1550		[1]
Al-Doped BP	doping	1550		[2]
Ag_2CrO_4/N-GQDs@g-C_3N_4	surface coating	1000		[3]
TiO_2@Perylene Diimide	surface coating	200–1000		[4]
Cu/G	surface coating	300–1800		[5]
WS_2@Cu	LSPR	1200		[6]
[ZnS–CdS–Cu_{2x}S]–ZnS	LSPR	900–1800		[7]
Bi/BiOBr:Yb^{3+},Er^{3+}	defects	980		[8]
[{MII$(MQ)_2$}{FeIII$(CN)_6$}]Cl·3H_2O	single component	355–2400		[9]
$CuWO_4$	single component	200–2100		[10]
Si	single component	200–1100	✓	[11]
InGaAs	single component	900–1700	✓	[12]
CdTe	single component	820	✓	[13]
$MAPbBr_3$	single component	350–600	✓	[14]
$[tmb^{\bullet+}]_4[Bi_4I_{16}]^{4-}$·7tmb (**1**)	single component	380–2200	✓	This work

References

[1] Y. Xu, C. Liu, C. Guo, Q. Yu, W. Guo, W. Lu, X. Chen, L. Wang, K. Zhang, *Nano Energy* **2020**, 70.

[2] Y. Liu, Y. Cai, G. Zhang, Y. W. Zhang, K. W. Ang, *Adv. Funct. Mater.* **2017**, 27.

[3] C. Feng, Y. Deng, L. Tang, G. Zeng, J. Wang, J. Yu, Y. Liu, B. Peng, H. Feng, J. Wang, *Appl. Catal. B Environ.* **2018**, 239, 525-536.

[4] W. Wei, Y. Zhu, *Small* **2019**, 15, e1903933.

[5] J. Xu, F. Xu, M. Qian, Z. Li, P. Sun, Z. Hong, F. Huang, *Nano Energy* **2018**, 53, 425-431.

[6] X. Xu, F. Luo, W. Tang, J. Hu, H. Zeng, Y. Zhou, *Adv. Funct. Mater.* **2018**, 28.

[7] T. T. Zhuang, Y. Liu, Y. Li, Y. Zhao, L. Wu, J. Jiang, S. H. Yu, *Angew. Chem. Int. Ed.* **2016**, 55, 6396-6400.

[8] Y. Li, Z. Wu, T. Liu, Z. Song, Y. Zhang, *Sol. RRL* **2021**, 5.

[9] X. Q. Yu, C. Sun, B. W. Liu, M. S. Wang, G. C. Guo, *Nat. Commun.* **2020**, 11, 1179.

[10] Z. Lin, W. Li, G. Yang, *Appl. Catal. B Environ.* **2018**, 227, 35-43.

[11] Q. Looker, M. G. Wood, A. Miceli, M. Niraula, K. Yasuda, J. L. Porter, *Rev. Sci. Instrum.* **2020**, 91, 023509.

[12] M. D. C. Whitaker, G. Lioliou, A. B. Krysa, A. M. Barnett, *Mater. Res. Express* **2020**, 7.

[13] Z. Y. He, C. M. Campbell, M. B. Lassise, Z. Y. Lin, J. J. Becker, Y. Zhao, M. Boccard, Z. Holman, Y. H. Zhang, *Appl. Phys. Lett.* **2016**, 109.

[14] H. Wei, Y. Fang, P. Mulligan, W. Chuirazzi, H. H. Fang, C. Wang, B. R. Ecker, Y. Gao, M. A. Loi, L. Cao, J. Huang, *Nat. Photonics* **2016**, 10, 333-339.
